# Associations Between Dietary Iron, SNP rs2794720, and Metabolic Syndrome Risk in Chinese Males and Females: A Community-Based Study in a Chinese Metropolis

**DOI:** 10.3390/nu17203185

**Published:** 2025-10-10

**Authors:** Zihan Hu, Hongwei Liu, Zhengyuan Wang, Jiajie Zang, Fan Wu, Zhenni Zhu

**Affiliations:** 1 School of Public Health, Fudan University, Shanghai 200437, China; zhhu24@m.fudan.edu.cn (Z.H.); hwliu23@m.fudan.edu.cn (H.L.); 2 Division of Health Risk Factors Monitoring and Control, Shanghai Municipal Center for Disease Control and Prevention, Shanghai 201107, China; wangzhengyuan@scdc.sh.cn (Z.W.); zangjiajie@scdc.sh.cn (J.Z.)

**Keywords:** metabolic syndrome, dietary iron, rs2794720, sex differences, genetic susceptibility

## Abstract

**Background:** Metabolic syndrome, a cardiovascular risk cluster, is recognized as a global health priority influenced by gene–diet interactions. The rs2794720 polymorphism has not been previously reported in relation to metabolic syndrome. This study examined the associations between dietary iron, SNP rs2794720, and metabolic syndrome in Chinese metropolitan population, with a focus on sex-specific and genotype-specific effects. **Methods:** A community-based cross-sectional study enrolled 2639 adults (1254 males, 1385 females) from Shanghai, China. Anthropometric measurements, laboratory analyses, and genotyping for the participants were performed. Dietary assessment utilized the 3-day 24 h dietary recall method. Metabolic syndrome was identified by the presence of at least three out of five metabolic abnormalities according to the NCEP—ATP III criteria. **Results:** After adjusting for confounders, in males, metabolic syndrome risk was associated with dietary iron (*p* = 0.002) but not with rs2794720 (*p* = 0.731). In females, metabolic syndrome risk was associated with rs2794720 (*p* = 0.014) and dietary iron (*p* = 0.016), with a significant interaction observed between rs2794720 and dietary iron (*p* = 0.047). Stratified by rs2794720, among females lacking the C allele, there was a linear trend between dietary iron and metabolic syndrome risk (*p* = 0.048). Compared to the reference group (lowest-intake GG homozygotes), the Q2–Q4 Ors (95% CI) were 5.31 (1.08, 39.52), 5.50 (1.16, 40.28), and 8.40 (1.80, 41.44)), while the major allele carriers did not show this trend (*p* = 0.704); compared to the reference group, the Q1–Q4 ORs(95% CI) were 6.13 (1.68, 39.66), 7.53 (2.06, 48.86), 8.10 (2.20, 52.60), and 7.84 (2.07, 51.70)). **Conclusions:** Our study first identified rs2794720 as a novel SNP associated with metabolic syndrome in Chinese females. The association between dietary iron and metabolic syndrome risk was unique to GG homozygotes (the minority), whereas CC/CG genotypes (the majority) showed no such association.

## 1. Introduction

Metabolic syndrome (MetS), characterized by a cluster of cardiometabolic abnormalities including central obesity, hypertension, dyslipidemia, and hyperglycemia [1], has emerged as a global public health challenge, with an estimated prevalence exceeding 30% nationally [2] and reaching 35% in Chinese urban populations [3]. This pathophysiological precursor not only predisposes individuals to type 2 diabetes and cardiovascular morbidity but also imposes escalating socioeconomic burdens on healthcare systems [4], particularly in rapidly urbanizing regions like Shanghai, where lifestyle transitions have exacerbated dietary imbalances and sedentary behaviors [5].

The etiology of MetS is multifactorial, involving complex interactions between environmental factors (e.g., diet, physical inactivity) and genetic susceptibility [6]. Among modifiable risk factors, dietary components such as iron intake have garnered increasing attention due to their dual role in metabolic homeostasis [7]. In urban Chinese populations, dietary iron patterns are characterized by a shift from plant-based non-heme iron to increasing heme iron from red meat and poultry, creating a distinct exposure profile compared to Western populations [8]. Whereas physiological iron bioavailability is indispensable for hemoglobin synthesis and metalloenzyme catalysis, disrupted iron homeostasis manifesting as hepatic iron overload has been mechanistically linked to mitochondrial oxidative damage, insulin receptor substrate-1 (*IRS-1*) serine phosphorylation, and NF-κB-mediated chronic low-grade inflammation—three cardinal pathomechanisms driving MetS progression [9,10].

Additionally, significant differences in sex and genotype existed with regard to the risk of developing MetS due to elevated iron levels. Research had consistently demonstrated that males were more susceptible to an increased risk of MetS when exposed to higher dietary iron compared to females [11]. Furthermore, different genotypes were linked to varying dietary iron fluctuation sensitivities [6,12]; some individuals may still have an increased risk of MetS due to genetic factors, which makes it crucial to identify genetic subgroups that are most responsive to dietary interventions.

Homeostatic Iron Regulator (*HFE*), located on human chromosome 6p21.3, is a central regulator of systemic iron metabolism [13]. It encodes a transmembrane protein that interacts with transferrin receptor 2 (*TFR2*) to modulate the expression of hepcidin [14], the master hormone governing iron absorption, recycling, and storage [15]. Dysregulation of *HFE* disrupts iron homeostasis, leading to pathological iron overload in hereditary hemochromatosis (HH) and contributing to metabolic disorders [16,17].

The SNP rs2794720, located near the *HFE* gene, has been implicated in iron metabolism pathways; however, its functional relevance and interaction with dietary factors remain unexplored. A critical void persists in understanding how rs2794720 variants mediate iron-induced metabolic dysregulation in Chinese populations, where traditional plant-based iron sources and unique *HFE* haplotype clusters may create distinct gene–environment interaction landscapes that modulate MetS susceptibility.

To address these gaps, we focused on the SNP rs2794720 due to its proximity to the *HFE* gene—a central regulator of systemic iron metabolism—which distinguishes it from other iron-related SNPs and suggests a unique potential to modulate iron-metabolism interactions. We conducted a community-based study in Shanghai, China, leveraging detailed dietary assessments and genotyping data. The objective of this study was to investigate the sex-specific associations between dietary iron, rs2794720 polymorphism, and the risks of MetS. We hypothesize that there is a positive correlation between dietary iron and the risk of MetS, and that rs2794720 may modify the association between dietary iron and MetS, with potential sex differences.

## 2. Materials and Methods

### 2.1. Study Population

**Figure 1:**
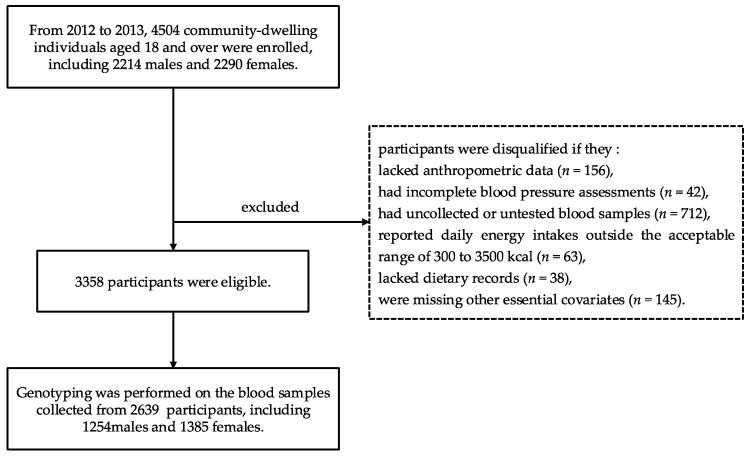
Flow chart of the study participants.

Based on the Shanghai Diet and Health Survey (SDHS), conducted between 2012 and 2013, a comprehensive random sampling strategy was implemented to enroll 4504 community-dwelling adults aged 18 years or older (2214 males and 2290 females) from diverse regions of Shanghai, China. Following enrollment, household surveys and blood sample collections were completed for all participants. Exclusion criteria included missing anthropometric measurements (*n* = 156), incomplete blood pressure data (*n* = 42), unavailable or untested blood samples (*n* = 712), daily energy intake outside the range of 300–3500 kcal (*n* = 63), absence of dietary records (*n* = 28), or incomplete essential covariate information (*n* = 145). After applying these exclusions, a final sample of 3358 eligible individuals was included in the study. Among these, genotyping for the SNP rs2794720 was successfully conducted in 2639 participants (1254 males and 1385 females) (Figure 1).

### 2.2. Dietary Assessment

A comprehensive dietary assessment was conducted to capture detailed food consumption patterns, covering a wide range of items including staples, side dishes, snacks, fruits, alcoholic and non-alcoholic beverages, as well as nutritional supplements. This assessment utilized the 3-day 24 h dietary recall method, which included two weekdays and one weekend day, chosen for its superior accuracy in evaluating iron intake compared to food frequency questionnaires (FFQ) [18]. The data collection was meticulously executed by qualified public health professionals from local community health service centers.

In addition, the weights of various primary condiments, such as edible oils, salt, and monosodium glutamate (MSG), were recorded at both the beginning and end of the 3-day survey period. The daily average intake of energy and iron for each individual was precisely calculated using the Chinese Food Composition database as the authoritative reference. It is important to note that iron from dietary supplements was excluded from the total dietary iron calculation, given the limited use of iron supplements among the Chinese population [19].

### 2.3. Anthropometric and Laboratory Measurements and Genotyping

Between 2012 and 2013, anthropometric measurements, comprising waist circumference and resting blood pressure, were performed by trained personnel at local community health centers. Concurrently, fasting venous blood samples were collected from participants after a 12 h overnight fast by trained investigators and were promptly processed for biochemical analyses. All laboratory analyses were conducted at the Shanghai Municipal Center for Disease Control and Prevention. For genotyping, white blood cells were immediately stored at −80 °C following collection during the 2012–2013 fieldwork to ensure cellular integrity. In 2018, DNA was extracted from these stored cells, and genotyping was performed for the single nucleotide polymorphism (SNP) rs2794720. Detailed information regarding the specific instruments and methods employed is provided in Table S1.

### 2.4. Metabolic Syndrome Definition

According to the US National Cholesterol Education Program Adult Treatment Panel III (NCEP-ATP III) criteria designed for Asian populations [20], MetS was identified by the presence of at least three out of the following five metabolic abnormalities: Firstly, an increased waist circumference, which was defined as ≥90 cm for men and ≥80 cm for women. Secondly, elevated triglyceride levels (≥150 mg/dL) or the use of medications for treating hypertriglyceridemia. Thirdly, a decrease in high-density lipoprotein cholesterol (HDL-C), with levels below 40 mg/dL for men and below 50 mg/dL for women, or being on medication to address low HDL-C. Fourthly, raised blood pressure, indicated by a systolic blood pressure of ≥130 mmHg and/or a diastolic blood pressure of ≥85 mmHg, or the use of antihypertensive drugs. Lastly, increased fasting glucose levels (≥100 mg/dL) or the use of medications for managing hyperglycemia.

### 2.5. Potential Confounders

The potential confounders were obtained through an interviewer-conducted survey. The survey collected data on age, sex, household income, education level, dietary energy intake, intentional physical activity, smoking status, and alcohol consumption. Among these variables, age, education level, and dietary energy intake were treated as continuous variables, while the remaining variables were considered categorical variables.

### 2.6. Statistical Analysis

We presented the distribution of covariates across sex using means (standard deviation, SD) and percentages, with between-group differences evaluated via chi-square tests for categorical variables and t-tests for continuous variables. Logistic regression models were utilized to examine the relationships between MetS risk and its components, and dietary iron as well as rs2794720. The models calculated odds ratios (ORs) and 95% confidence intervals (CIs), with the occurrence of MetS serving as the dependent variable. Independent variables included the presence of at least one C allele at rs2794720 (coded as a binary variable: 1 if present, 0 if absent) and quartiles of dietary iron. Additionally, the relative excess risk due to interaction (RERI) was computed to assess potential additive-scale effect modification. To address potential bias from sparse data in subgroup analyses, particularly the elevated odds ratios observed in some strata, we performed bootstrap resampling with Firth’s bias-reduced logistic regression. We conducted 1000 bootstrap replicates, calculating odds ratios and 95% confidence intervals using the percentile method. The Firth-penalized likelihood approach provides finite estimates even when maximum likelihood estimation fails due to separation, ensuring more reliable effect size estimates in small subgroups. All analyses were conducted using R version 4.4.2 (accessed on 31 October 2024), with statistical significance determined by a two-tailed *p*-value of less than 0.05. The “epiR” package was employed to evaluate additive interaction effects. The “logistf” package was employed to perform bootstrap resampling with Firth’s bias-reduced logistic regression.

## 3. Results

### 3.1. Participants’ Characteristics

As presented in Table 1, the study involved 2639 Chinese adults, with males accounting for 47.5% (*n* = 1254) and females for 52.4% (*n* = 1385). MetS was identified in 24.1% of the participants, with MetS component details in Table S2. The average daily iron intake was 19.3 ± 15.6 mg, with significant sex differences observed: males consumed 21.5 ± 19.3 mg compared to 17.3 ± 10.6 mg among the females. Males and females also differed in household income, education level, dietary energy intake, intentional physical activity, smoking status, and alcohol consumption.

### 3.2. Genotypes of the rs2794720

The distribution of rs2794720 genotypes among participants showed that 54.1% carried the CC genotype, 36.1% had the CG genotype, and 9.8% possessed the GG genotype, with no significant sex difference (*p* = 0.955), as detailed in Table 2. The minor allele frequency (MAF) of the G allele was 27.9%, with no significant difference between males (28.6%) and females (27.3%) (*p* = 1.000).

### 3.3. Associations of MetS Risk Stratified with Dietary Iron and the rs2794720

After adjusting for age, sex, income, education, physical exercise, smoking, alcohol consumption, and total dietary energy intake, our analysis identified a significant correlation between dietary iron and an increased risk of MetS (Table 3). Specifically, compared to the lowest quartile of dietary iron (<12.69 mg/day), the ORs with 95% CIs for MetS risk were 1.28 (0.96, 1.70) in Q2 (12.69–16.50 mg/day), 1.46 (1.09, 1.97) in Q3 (16.50–19.90 mg/day), and 1.58 (1.12, 2.24) in Q4 (≥21.73 mg/day). Notably, no significant association was observed between the SNP rs2794720 and MetS risk among all the participants.

Further stratification by sex revealed a positive correlation between dietary iron and MetS risk in males. Compared to the males in the lowest quartile (<14.13 mg/day), the ORs (95% CIs) were 1.29 (0.82, 2.05) in Q2 (14.13–17.70 mg/day), 1.89 (1.19, 3.04) in Q3 (17.70–23.49 mg/day), and 2.10 (1.24, 3.58) in Q4 (≥23.49 mg/day). In contrast, no such correlation was found in females. However, the SNP rs2794720 was associated with MetS in the females, with ORs (95% CIs) of 1.57 (1.01, 2.54) for major allele carriers compared to non-carriers, suggesting a potential sex-specific effect of this genetic variant on MetS risk.

In addition to the main effects, we explored the interaction between SNP rs2794720 and dietary iron. In the females, we observed a significant multiplicative interaction between SNP rs2794720 and dietary iron, indicating a joint effect on the risk (OR = 0.64 (0.41, 0.98)). Conversely, no multiplicative interaction was detected between these two factors in the male participants (OR = 1.19 (0.75, 1.89)). These findings are summarized in Table 4. We also conducted an analysis of additive interaction, but no significant interaction was observed for the increased risk of MetS (Table S3). We also investigated the associations and interactions between the components of MetS and dietary iron and rs2794720, where both increased blood pressure and elevated triglycerides showed statistically significant associations and interactions (Table S4). Furthermore, we examined the associations of heme iron and non-heme iron with MetS risk and their interactions with rs2794720. The analysis revealed significant associations and interactions for non-heme iron (Table S5), but not for heme iron (Table S6).

### 3.4. Associations Between Dietary Iron and Risk of MetS Stratified by rs2794720

**Figure 2:**
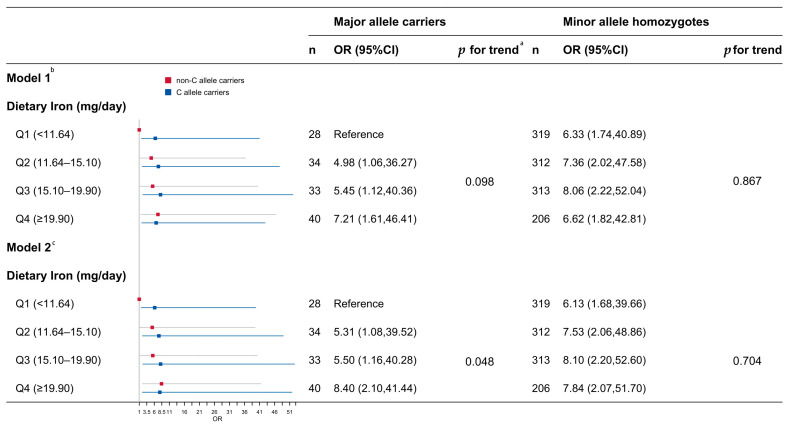
Associations between dietary iron and the risk of MetS, stratified by the presence of the C allele of the SNP rs2794720 among the female participants. Note: ^a^ The *p* value for the trend was examined using the medians in each quartile of dietary iron. ^b^ Model 1 was adjusted for age, sex, income, education, intentional physical exercise, smoking status, alcohol use, and total dietary energy. ^c^ Model 2, in addition to adjusting for the aforementioned covariates, also includes the interaction term between SNP and dietary iron.

Given that associations were only observed in the female participants, and significant *p*-values from previous interaction analyses indicated heterogeneity within subgroups, subsequent analyses were conducted exclusively on the females to further stratify and investigate this heterogeneity. Within this context, the C allele at rs2794720 was identified as the risk allele. After adjusting for confounding factors such as age, income, years of education, self-reported exercise habits, smoking status, alcohol consumption, and total dietary energy intake (Model 2), a significant trend remained between dietary iron and the risk of MetS in individuals lacking the C allele (*p* for trend = 0.048). In contrast, this relationship was not observed in individuals carrying the C allele (*p* for trend = 0.704). When comparing the OR values of the quartiles of dietary iron with the reference group (the subgroup with the lowest intake among minor allele homozygotes), the ORs for the risk of MetS were as follows: for Q2, 5.31 (1.08, 39.52); for Q3, 5.50 (1.16, 40.28); and for Q4, 8.40 (1.80, 41.44) (Figure 2). We also explored the relationships between the components of MetS and dietary iron (Figures S1–S5) and the associations between MetS and non-heme iron (Figure S6), stratified by rs2794720. The results revealed trends consistent with those for total dietary iron.

### 3.5. Sensitivity Analysis

To assess the robustness of our primary findings, we performed bootstrap validation with Firth correction. The results indicated that while the direction and statistical significance of all associations remained consistent, there were notable differences in point estimates. After adjusting for confounding factors—including age, income, years of education, self-reported exercise habits, smoking status, alcohol consumption, and total dietary energy intake (Model 2)—the bootstrap-derived ORs were systematically higher than the original maximum likelihood estimates. When comparing the OR values of the quartiles of dietary iron with the reference group (the subgroup with the lowest intake among minor allele homozygotes), the ORs for the risk of MetS were as follows: for Q2, 8.29 (1.11, 38.34); for Q3, 8.55 (1.13, 39.35); and for Q4, 13.16 (1.87, 56.78) (Table S7).

## 4. Discussion

Our study was the first to identify rs2794720 as a Chinese female-specific factor in the associations between dietary iron and MetS. The rs2794720 variant, located 2KB upstream of the *HFE* gene [21], a central regulator of systemic iron metabolism, is a non-coding transcript variant that may interfere with the transcriptional initiation of *HFE* [22]. This disruption can impair the hepcidin-*TFR2* interaction, compromise hepatic iron sensing, and lead to increased systemic iron accumulation alongside an insufficient hepcidin response [23,24], which in turn contributes to the development of the MetS. Unlike Western populations where *HFE* C282Y/H63D mutations dominate iron-related disorders [25,26,27], rs2794720 may represent a population-specific variant influencing iron metabolism in Chinese female individuals. This is consistent with its allele frequency, which is highest in East Asian populations (~28%) compared to European (~15%), African (~8%), and South Asian (~12%) populations (1000 Genomes Project) [28], underscoring its potential unique role in Chinese female individuals.

Remarkably, rs2794720 might create a genetically vulnerable female subpopulation with amplified iron sensitivity. In females, the absence of a direct association between dietary iron and MetS may stem from estrogen-mediated suppression of hepcidin, which regulates hepatic iron pathways, with this effect amplified by physiological iron cycling through menstrual blood loss [29]. However, the interaction between rs2794720 and dietary iron in females implies that genetic susceptibility amplifies the metabolic consequences of iron intake in this subgroup. The mechanism of sex-specific divergence may be related to the estrogen–hepcidin metabolic pathway regulated by the *HFE* gene [30,31]. The *HFE* gene enhances hepcidin expression through an estrogen-mediated pathway in individuals without the rs2794720-C allele. In female rs2794720 minor allele homozygotes, the *HFE* gene enhanced hepcidin expression through an estrogen-mediated pathway, which inhibited iron absorption, thereby reducing systemic iron overload [32,33], but increasing sensitivity to genetic perturbations in the iron metabolism pathway.

In contrast, males demonstrated susceptibility to classical iron overload pathophysiology, with their dose-dependent MetS risk from elevated iron intake confirming established iron–metabolic dysfunction pathways [11]. Excessive dietary iron might have induced systemic iron overload (IO), which triggers oxidative stress-mediated mitochondrial damage and impairs *IRS-1* signaling through serine phosphorylation, fostering insulin resistance and hepatic inflammation [34,35]—key drivers of MetS pathogenesis [36]. Supporting the specificity of the Chinese diet, additional analyses showed that the observed link between total iron intake and MetS risk was attributable to non-heme iron, the predominant source in this population [8]. In contrast to Western studies that establish heme iron as a risk factor [37], we observed no significant association, a result that may be attributed to its considerably lower intake within our study population.

Our results are the first to report the relationships between the rs2794720 polymorphism, dietary iron, and MetS risk, identifying rs2794720 as a sex-specific modifier of the iron–MetS association in Chinese females and delineating a dietary iron-sensitive subpopulation within this group. Mechanistically, the dual impact of rs2794720 on iron metabolism and MetS pathogenesis warrants further investigation. Future research should prioritize functional studies to validate its regulatory role in *HFE* or hepcidin expression (e.g., using luciferase reporter assays could determine if the rs2794720 variant alters promoter activity or gene expression of *HFE*). Clinically, our findings challenge the conventional “one-size-fits-all” approach. However, the low prevalence of GG genotype individuals limits the broader applicability of these findings to public health policy. While precision nutrition approaches [38] could benefit high-risk subgroups, the limited population size of minor allele female homozygotes diminishes targeted interventions in females. The low frequency of genotype-driven iron sensitivity does not justify revising current guidelines. Existing strategies, such as limiting red meat consumption [39,40] remain broadly effective for MetS prevention. Given the current evidence base, public health priorities should primarily emphasize these established interventions in male populations, where population-level implementation demonstrates greater feasibility.

Several limitations warrant consideration. First, the cross-sectional design precludes causal inference, as reverse causation (e.g., dietary shifts away from iron-rich foods following MetS onset) cannot be excluded. Longitudinal studies are needed for further clarification. Second, the biological function of rs2794720 remains uncharacterized. Future studies are warranted not only to validate its regulatory effects on *HFE* or hepcidin expression but also to explore the potential synergistic contributions of multiple SNPs to MetS susceptibility. Third, the subgroup analyses among females stratified by the rs2794720 C allele were underpowered due to small sample sizes, potentially yielding non-significant results (such as after FDR correction) and wide confidence intervals, necessitating validation in larger future studies. Finally, the Shanghai-centric sample limits generalizability, necessitating replication in multiethnic populations to validate the observed sex–genotype interactions.

## 5. Conclusions

In this community-based study, we first identified rs2794720 as a novel SNP associated with MetS in the Chinese metropolitan females. GG homozygotes (the genetic minority subgroup) demonstrated a significant association between dietary iron and risks of MetS, whereas the majority, major allele carriers, showed stable MetS risk across dietary iron quartiles. Although this was a novel discovery, it remains consistent with previous studies that females were generally insensitive to iron intake in relationto MetS risk.

## Figures and Tables

**Table 1:** Characteristics of the participants, stratified by sex.

	Total	Male	Female	*p*
*n* (%)	2639 (100.0)	1254 (47.5)	1385 (52.4)	
Age (%)				
15−44 years	786 (29.8)	363 (29.0)	423 (30.6)	0.560
45–59 years	1019 (38.7)	484 (38.6)	535 (38.7)	
60+ years	831 (31.5)	406 (32.4)	425 (30.7)	
Annual Household Income (%)				
Above average level (RMB > 60,000) ^a^	120 (4.6)	60 (4.8)	60 (4.3)	0.541
Average level (RMB 30,000–59,999)	1493 (56.6)	703 (56.1)	790 (57.1)	
Below average level (RMB < 30,000)	812 (30.8)	397 (31.7)	415 (30.0)	
No answer	212 (8.0)	93 (7.4)	119 (8.6)	
Years of Education, years (SD) ^b^	9.5 (4.5)	10.1 (4.0)	8.9 (4.8)	<0.001
Intentional Physical Exercise (%)				
Yes	1958 (74.4)	930 (74.5)	1028 (74.4)	0.973
no	672 (25.6)	318 (25.5)	354 (25.6)	
Smoking Status (%)				
Never smoked	1850 (70.2)	485 (38.7)	1365 (98.6)	<0.001
Former smoker	142 (5.4)	137 (10.9)	5 (0.4)	
Current smoker	644 (24.4)	630 (50.3)	14 (1.0)	
Alcohol Use (%)				
Lifetime abstainers	1980 (79.7)	716 (62.2)	1264 (94.8)	<0.001
Nonheavy drinkers	397 (16.0)	336 (29.2)	61 (4.6)	
Infrequent heavy drinkers	32 (1.3)	28 (2.4)	4 (0.3)	
Frequent heavy drinkers	75 (3.0)	71 (6.2)	4 (0.3)	
Dietary Intake				
Energy, kcal/day (SD)	1763.3 (852.6)	1941.6 (915.4)	1602.0 (756.4)	<0.001
Iron Intake				
Total iron, mg/day (SD)	19.4 (16.4)	21.8 (21.0)	17.3 (10.4)	<0.001
Heme iron, mg/day (SD)	1.6 (1.4)	1.7 (1.5)	1.5 (1.2)	<0.001
Non-heme iron, mg/day (SD)	17.9 (16.0)	20.1 (20.5)	15.9 (9.8)	<0.001
Metabolic Syndrome				
Yes	635 (24.1)	273 (21.8)	362 (26.1)	0.010
no	2004 (75.9)	981 (78.2)	1023 (73.9)	

Note: ^a^ RMB, Renminbi, the currency of P.R. China; ^b^ SD, standard deviation.

**Table 2:** Genotypes of the rs2794720 in the study participants.

	Frequency (%)			
	Total	Male	Female	*p* Value ^f^
Genotype				
Major allele carriers ^a^	2380 (90.2)	1130 (90.1)	1250 (90.2)	0.955
CC ^b^	1427 (54.1)	661 (52.7)	766 (55.3)	
CG ^c^	953 (36.1)	469 (37.4)	484 (34.9)	
Minor allele homozygotes				
GG ^d^	259 (9.8)	124 (9.9)	135 (9.7)	
MAF ^e^				
G	27.9	28.6	27.3	1.000

Note: ^a^ Participants who carried at least one C allele on the rs2794720, including CC and CG; ^b^ CC, double C allele; ^c^ CG, one C allele and one G allele; ^d^ GG, double G allele; ^e^ MAF, minor allele frequency; ^f^ the *p*-value for testing whether there was a difference in the proportion of the major allele carriers and MAF of C between the males and females was determined using the Chi-squared test.

**Table 3:** ORs (95% CI) for risk of MetS according to dietary iron and the SNP rs2794720 in the participants, stratified by sex ^a^.

	Model 1 ^b^		Model 2 ^c^	
	OR (95% CI) ^d^	*p* Values	OR (95% CI)	*p* Values
Total (Sex-Adjusted)				
Dietary iron				
Q1 (<12.69 mg/day)	Reference		Reference	
Q2 (12.69–16.50 mg/day)	1.25 (0.96, 1.63)	0.101	1.28 (0.96, 1.70)	0.090
Q3 (16.50–19.90 mg/day)	1.42 (1.09, 1.85)	0.010	1.46 (1.09, 1.97)	0.013
Q4 (≥21.73 mg/day)	1.39 (1.07, 1.82)	0.015	1.58 (1.12, 2.24)	0.010
rs2794720				
Minor allele homozygotes	Reference		Reference	
Major allele carriers	1.26 (0.92, 1.74)	0.156	1.31 (0.95, 1.85)	0.108
Male				
Dietary iron				
Q1 (<14.13 mg/day)	Reference		Reference	
Q2 (14.13–17.70 mg/day)	1.24 (0.82, 1.89)	0.313	1.29 (0.82, 2.05)	0.273
Q3 (17.70–23.49 mg/day)	1.78 (1.21, 2.66)	0.004	1.89 (1.19, 3.04)	0.007
Q4 (≥23.49 mg/day)	1.83 (1.24, 2.72)	0.003	2.10 (1.24, 3.58)	0.006
rs2794720				
Minor allele homozygotes	Reference		Reference	
Major allele carriers	0.94 (0.61, 1.49)	0.774	0.92 (0.57, 1.53)	0.733
Female				
Dietary iron				
Q1 (<11.64 mg/day)	Reference		Reference	
Q2 (11.64–15.10 mg/day)	1.26 (0.88, 1.81)	0.211	1.33 (0.91, 1.96)	0.140
Q3 (15.10–19.90 mg/day)	1.36 (0.95, 1.95)	0.091	1.43 (0.97, 2.13)	0.074
Q4 (≥19.90 mg/day)	1.18 (0.82, 1.70)	0.362	1.46 (0.91, 2.35)	0.120
rs2794720				
Minor allele homozygotes	Reference		Reference	
Major allele carriers	1.59 (1.02, 2.55)	0.047	1.57 (1.01, 2.54)	0.048

Note: ^a^ The presence of the C allele of rs2794720 is encoded as 0 for non-existence and 1 for existence. Dietary iron is categorized into four groups based on quartiles, each including its lower boundary, with Q1 serving as the reference. ^b^ Model 1 was adjusted for age and sex. ^c^ Model 2 was adjusted for age, sex, income, education, intentional physical exercise, smoking status, alcohol use, and total dietary energy. ^d^ The OR (95% CI) for dietary iron represents the risk of MetS within the current range of dietary iron compared to the reference group. The OR (95% CI) for rs2794720 indicates the multiple increase in the risk of MetS for individuals carrying the C allele compared to those who do not carry the C allele.

**Table 4:** Effect heterogeneity analysis on the association between dietary iron, rs2794720, and their interactions with MetS, stratified by sex ^a^.

	Model 1 ^b^		Model 2 ^c^		*p*_LR_ ^e^
	OR (95% CI) ^d^	*p* Values	OR (95% CI)	*p* Values	
Total (Sex-Adjusted)					
Dietary iron	1.16 (1.04, 1.30)	0.007	1.29 (0.95, 1.76)	0.099	0.465
rs2794720	1.31 (0.95, 1.84)	0.113	1.77 (0.75, 4.41)	0.206	
Interaction term			0.89 (0.65, 1.21)	0.466	
Male					
Dietary iron	1.30 (1.10, 1.54)	0.002	1.11 (0.70, 1.75)	0.664	0.463
rs2794720	0.92 (0.57, 1.53)	0.731	0.60 (0.18, 2.16)	0.411	
Interaction term			1.19 (0.75, 1.89)	0.463	
Female					
Dietary iron	1.13 (0.98, 1.31)	0.104	1.70 (1.12, 2.67)	0.016	0.042
rs2794720	1.57 (1.00, 2.53)	0.055	5.31 (1.49, 22.16)	0.014	
Interaction term			0.64 (0.41, 0.98)	0.047	

Note: ^a^ The presence of the C allele of rs2794720 is coded as 0 for absence and 1 for presence. To calculate the interaction, dietary iron is included as a continuous variable when calculating the *p* interaction. ^b^ Model 1 was adjusted for age, sex, income, education, intentional physical exercise, smoking status, alcohol use, and total dietary energy. ^c^ Model 2, in addition to adjusting for the aforementioned covariates, also includes the interaction term between SNP and dietary iron. ^d^ The ORs (95% CI) for dietary iron reflect the increased risk of MetS associated with a 25% increment in dietary iron relative to the reference group. The ORs (95% CI) for rs2794720 denote the fold increase in the risk of MetS among individuals carrying the C allele compared to those who do not carry the C allele. The interaction term suggests a potential interaction between dietary iron and rs2794720 in influencing the risk of MetS. ^e^ The *p* value for the likelihood ratio test results for Model 1 and Model 2.

## Data Availability

The data presented in this study are available on request from the corresponding author due to restrictions specified in the ethical approval for this research, which prohibit the public sharing of participant data.

## Supplementary Materials

The following supporting information can be downloaded at https://www.mdpi.com/article/10.3390/nu17203185/s1, Table S1: Detailed information regarding the specific instruments and methods; Table S2: MetS component of the participants, stratified by sex; Table S3: Additive interaction results (95% CI) for dietary iron intake and the SNP rs2794720 among the female participants; Table S4: Effect Heterogeneity Analysis on the Association between Dietary Iron, rs2794720, and Their Interaction with Incident Outcomes, Stratified by sexa; Table S5: ORs (95% CI) and multiplicative interaction for MetS risk by non-heme iron and rs2794720, stratified by sex; Table S6: ORs (95% CI) and multiplicative interaction for MetS risk by heme iron and rs2794720, stratified by sex; Table S7: Bootstrap-Validated ORs (95% CI) for the Association between Dietary Iron Intake and Metabolic Syndrome Risk, Stratified by Genotype; Figures S1–S5: The association between dietary iron and risk of MetS component stratified by C allele presence of the SNP rs2794720 among the female participants; Figure S6: The association between non-heme iron and risk of MetS stratified by C allele presence of the SNP rs2794720 among the female participants.

## Abbreviations

MetS	Metabolic Syndrome
IRS-1	Insulin Receptor Substrate-1
HFE	Homeostatic Iron Regulator
TFR2	Transferrin Receptor 2
HH	Hereditary Hemochromatosis
SDHS	Shanghai Diet and Health Survey
FFQ	Food Frequency Questionnaires
MSG	Monosodium Glutamate
SNP	Single Nucleotide Polymorphism
NCEP-ATP III	National Cholesterol Education Program Adult Treatment Panel III
HDL-C	High-Density Lipoprotein Cholesterol
SD	Standard Deviation
ORs	Odds Ratios
CIs	Confidence Intervals
RERI	Relative Excess Risk Due to Interaction
RMB	Renminbi, the currency of P.R. China
MAF	Minor Allele Frequency
IO	Iron Overload
