# Supplementary material for: Associations Between Dietary Iron, SNP rs2794720, and Metabolic Syndrome Risk in Chinese Males and Females: A Community-Based Study in a Chinese Metropolis
Source: Nutrients. 2025 Oct 10;17(20):3185. doi: 10.3390/nu17203185 (PMC12567378; doi:10.3390/nu17203185)
Supplement: Supplementary file 1 [file nutrients-17-03185-s001.zip › nutrients-3883379-supplementary.pdf]

Table S1: Detailed information regarding the specific instruments and methods

Measurement Category	Instrument/Reagent
Anthropometric Measurements	
Waist circumference	Graham-Field 1340-2 tape measure (precision ±1mm)
Blood pressure	Omron HEM-7071 electronic sphygmomanometer (Kyoto, Japan)
Laboratory Analyses	
Glucose/TG/HDL-C	HITACHI 7080 Analyzer with Wako reagents (Tokyo, Japan)
Genetic Analysis	
DNA extraction	TIANGEN DP705-02 Kit (magnetic bead method, Beijing, China)
SNP rs2794720 genotyping	Applied Biosystems 3730XL Analyzer with SNaPshot Multiplex System (Waltham, USA)
Data analysis	GeneMapper Software v4.0 (Thermo Fisher Scientific)

Table S2: MetS component of the participants, stratified by sex.

	**All**	**Male**	**Female**	***p***
Metabolic Syndrome Components				
Elevated blood pressure (%)				
Yes	1409(53.4)	721(57.5)	688(49.7)	<0.001
no	1230(46.6)	533(42.5)	697(50.3)	
Elevated triglycerides (%)				
Yes	713(27.0)	375(29.9)	338(24.4)	0.002
no	1926(73.0)	879(70.1)	1047(75.6)	
Elevated waist circumference (%)				
Yes	904(34.3)	329(26.2)	575(41.5)	<0.001
no	1735(65.7)	925(73.8)	810(58.5)	
Elevated fasting glucose (%)				
Yes	610(23.1)	320(25.5)	290(20.9)	0.006
no	2029(76.9)	934(74.5)	1095(79.1)	
Reduced HDL-C[a] (%)				
Yes	536(20.3)	184(14.7)	352(25.4)	<0.001
no	2103(79.7)	1070(85.3)	1033(74.6)	

NOTE: [a] HDL-C, High density lipoprotein cholesterol

Table S3: Additive interaction results (95% CI) for dietary iron intake and the SNP rs2794720 among the female participants.[1]

**Gene Site**	**RERI[2]**	**AP[3]**	**S[4]**
rs2794720	-0.23 (-1.12 ,0.66)	-0.04 (-0.20, 0.12)	0.95 (0.30, 1.60)

[1] C allele presence of rs2794720 was coded as 1 for presence and 0 for non-presence. [2] RERI, Relative Excess Risk due to Interaction. [3] AP, Attributable Proportion. [4] S, Synergy index.

Table S4: Effect Heterogeneity Analysis on the Association between Dietary Iron, rs2794720, and Their Interaction with Incident Outcomes, Stratified by sex[a]

	Model 1[b]		Model 2[c]		
	OR(95%CI)[d]	*p* values	OR(95%CI)	*p* values	p_{LR}[e]
Elevated blood pressure					
Dietary iron	1.03 (0.89,1.19)	0.691	1.97 (1.33,2.94)	0.001	
rs2794720	1.01 (0.66,1.55)	0.966	6.90 (2.17,22.57)	0.001	<0.001
Interaction term			0.49 (0.33,0.73)	0.001	
Elevated triglycerides					
Dietary iron	1.03 (0.89,1.19)	0.675	1.59 (1.06,2.44)	0.028	
rs2794720	1.23 (0.80,1.94)	0.359	4.46 (1.33,17.47)	0.022	0.023
Interaction term			0.62 (0.40,0.94)	0.027	
Elevated waist circumference					
Dietary iron	1.16 (0.99,1.36)	0.068	1.25 (0.80,1.98)	0.333	
rs2794720	1.54 (0.95,2.59)	0.092	1.91 (0.54,7.87)	0.340	0.104
Interaction term			0.92 (0.58,1.45)	0.727	
Elevated fasting glucose					
Dietary iron	1.08 (0.95,1.23)	0.260	1.40 (1.00,1.98)	0.055	
rs2794720	1.06 (0.73,1.56)	0.747	2.27 (0.85,6.33)	0.109	0.727
Interaction term			0.75 (0.53,1.06)	0.106	
Reduced HDL-C					
Dietary iron	1.05 (0.91,1.21)	0.508	1.49 (0.98,2.35)	0.072	
rs2794720	1.63 (1.04,2.64)	0.040	4.59 (1.30,19.63)	0.026	0.085
Interaction term			0.68 (0.43,1.05)	0.092	

NOTE: [a] The presence of the C allele of rs2794720 is coded as 0 for absence and 1 for presence. To calculate the interaction, dietary iron is included as a continuous variable when calculating the *p* interaction.

[b] Model 1 was adjusted for age, sex, income, education, intentional physical exercise, smoking status, alcohol use, and total dietary energy.

[c] Model 2, in addition to adjusting for the aforementioned covariates, also includes the interaction term between SNP and dietary iron.

[d] The OR (95% CI) for dietary iron reflect the increased risk of metabolic syndrome associated with a 25% increment in dietary iron relative to the reference group. The OR (95% CI) for rs2794720 denote the fold increase in the risk of metabolic syndrome among individuals carrying the C allele compared to those who do not carry the C allele. The interaction term suggests a potential interaction between dietary iron and rs2794720 in influencing the risk of metabolic syndrome.

[e] The *p* value for the likelihood ratio test results for Model 1 and Model 2.

Table S5: ORs (95% CI) and multiplicative interaction for MetS risk by non-heme iron and rs2794720, stratified by sex[a].

	Model 1 [b]			Model 2 [c]		
	OR [d]	p_{trend} [e]	p_{INTM} [f]	OR [d]	p_{trend}	p_{INTM}
All						
Dietary iron						
Q1 (<11.57)	Reference			Reference		
Q2 (11.57–15.13)	1.85 (0.73,4.90)	0.240		2.07 (0.78,5.77)	0.139	
Q3 (15.13–20.00)	1.77 (0.72,4.58)			1.83 (0.70,5.05)		
Q4 (≥20.00)	1.90 (0.75,5.02)		0.644	2.37 (0.87,6.80)		0.534
rs2794720						
Minor allele homozygotes	Reference	0.343		Reference	0.241	
Major allele carriers	1.67 (0.81,3.77)			1.85 (0.88,4.41)		
Male						
Dietary iron						
Q1 (<12.76)	Reference			Reference		
Q2 (12.76–16.22)	0.77 (0.23,2.55)	0.974		0.66 (0.17,2.50)	0.627	
Q3 (16.22–21.47)	0.82 (0.24,2.84)			0.87 (0.22,3.42)		
Q4 (≥21.47)	0.98 (0.28,3.43)		0.349	1.30 (0.31,5.36)		0.567
rs2794720						
Minor allele homozygotes	Reference	0.325		Reference	0.509	
Major allele carriers	0.55 (0.22,1.48)			0.53 (0.20,1.57)		
Female						
Dietary iron						
Q1 (<10.55)	Reference			Reference		
Q2 (10.55–13.81)	3.33 (0.74,18.01)	0.048		3.18 (0.70,17.28)	0.030	
Q3 (13.81–18.21)	3.24 (0.80,16.65)			3.47 (0.84,17.92)		
Q4 (≥18.21)	4.67 (1.21,23.33)		0.071	5.15 (1.28,26.52)		0.049
rs2794720						
Minor allele homozygotes	Reference	0.020		Reference	0.022	
Major allele carriers	4.53 (1.47,19.92)			4.45 (1.43,19.65)		

NOTE: [a] The presence of the C allele of rs2794720 is encoded as 0 for non-existence and 1 for existence. Dietary iron is categorized into four groups based on quartiles, each including its lower boundary, with Q1 serving as the reference. [b] Model 1 was adjusted for age and sex. [c] Model 2 was adjusted for age, sex, income, education, intentional physical exercise, smoking status, alcohol use, and total dietary energy. [d] The OR (95% CI) for dietary iron represents the risk of mets within the current range of dietary iron compared to the reference group. The OR (95% CI) for rs2794720 indicates the multiple increase in the risk of mets for individuals carrying the C allele compared to those who do not carry the C allele. [e] p_{trend}, the p value for the trend was examined using the medians in each quartile of dietary iron. [f] p_{INTM}, the p value of multiplicative interaction.

Table S6: ORs (95% CI) and multiplicative interaction for MetS risk by heme iron and rs2794720, stratified by sex [a].

	Model 1 [b]			Model 2 [c]		
	OR [d]	p_{trend} [e]	p_{INTM} [f]	OR [d]	p_{trend} [e]	p_{INTM} [f]
All						
Dietary iron						
Q1 (<0.82)	Reference					
Q2 (0.82–1.27)	0.96 (0.40,2.33)	0.755		0.99 (0.39,2.49)	0.822	
Q3 (1.27–1.91)	0.92 (0.39,2.17)			0.78 (0.31,1.96)		
Q4 (≥1.91)	1.17 (0.50,2.76)		0.908	1.19 (0.49,2.88)		0.906
rs2794720						
Minor allele homozygotes	Reference	0.516		Reference	0.454	
Major allele carriers	1.21 (0.65,2.38)			1.26 (0.67,2.54)		
Male						
Dietary iron						
Q1 (<0.93)						
Q2 (0.93–1.37)	0.43 (0.11,1.44)	0.300		0.30 (0.06,1.24)	0.190	
Q3 (1.37–2.00)	0.68 (0.23,1.96)			0.54 (0.17,1.71)		
Q4 (≥2.00)	0.43 (0.10,1.54)		0.136	0.36 (0.08,1.38)		0.140
rs2794720						
Minor allele homozygotes	Reference	0.136		Reference	0.140	
Major allele carriers	0.46 (0.20,1.09)			0.41 (0.17,1.03)		
Female						
Dietary iron						
Q1 (<0.75)						
Q2 (0.75–1.19)	1.75 (0.47,6.87)	0.229		1.86 (0.50,7.34)	0.196	
Q3 (1.19–1.80)	1.08 (0.23,4.83)			1.06 (0.23,4.77)		
Q4 (≥1.80)	2.36 (0.72,8.60)		0.215	2.61 (0.78,9.76)		0.234
rs2794720						
Minor allele homozygotes	Reference	0.061		Reference	0.071	
Major allele carriers	2.64 (1.02,8.24)			2.65 (1.02,8.32)		

NOTE: [a] The presence of the C allele of rs2794720 is encoded as 0 for non-existence and 1 for existence. Dietary iron is categorized into four groups based on quartiles, each including its lower boundary, with Q1 serving as the reference. [b] Model 1 was adjusted for age and sex. [c] Model 2 was adjusted for age, sex, income, education, intentional physical exercise, smoking status, alcohol use, and total dietary energy. [d] The OR (95% CI) for dietary iron represents the risk of mets within the current range of dietary iron compared to the reference group. The OR (95% CI) for rs2794720 indicates the multiple increase in the risk of mets for individuals carrying the C allele compared to those who do not carry the C allele. [e] p_{trend}, the *p* value for the trend was examined using the medians in each quartile of dietary iron. [f] p_{INTM}, the *p* value of multiplicative interaction.

Table S7: Bootstrap-Validated ORs (95% CI) for the Association between Dietary Iron Intake and Metabolic Syndrome Risk, Stratified by Genotype.

Dietary Iron (mg/day)	Model 1 [a]		Model 2 [b]	
	Original OR	Bootstrap OR	Original OR	Bootstrap OR
**Major allele carriers**				
**Q1 (<11.64)**	Reference		Reference	
**Q2 (11.64–15.10)**	4.98 (1.06,36.27)	8.37 (1.13,39.79)	5.31 (1.08,39.52)	8.29 (1.11,38.34)
**Q3 (15.10–19.90)**	5.45 (1.12,40.36)	7.64 (1.08,36.55)	5.50 (1.16,40.28)	8.55 (1.13,39.35)
**Q4 (≥19.90)**	7.21 (1.61,46.41)	10.84 (1.66,45.28)	8.40 (2.10,41.44)	13.16 (1.87,56.78)
**Minor allele homozygotes**				
**Q1 (<11.64)**	6.33 (1.74,40.89)	8.93 (1.89,33.94)	6.13 (1.68,39.66)	8.68 (1.80,33.70)
**Q2 (11.64–15.10)**	7.36 (2.02,47.58)	10.30 (2.17,40.88)	7.53 (2.06,48.86)	10.60 (2.25,42.00)
**Q3 (15.10–19.90)**	8.06 (2.22,52.04)	11.32 (2.32,43.79)	8.10 (2.20,52.60)	11.40 (2.25,42.26)
**Q4 (≥19.90)**	6.62 (1.82,42.81)	9.27 (1.93,34.64)	7.84 (2.07,51.70)	11.18 (2.12,43.06)

[a] Model 1 was adjusted for age, sex, income, education, intentional physical exercise, smoking status, alcohol use, and total dietary energy.

[b] Model 2, in addition to adjusting for the aforementioned covariates, also includes the interaction term between SNP and dietary iron.

Elevated blood pressure	non-C allele carriers		C allele carriers	
	OR (95%CI)	p trend[a]	OR (95%CI)	p trend
**Model 1**[b]				
**Dietary Iron (mg/day)**				
Q1 (<11.64)	Reference	0.018	3.66 (1.36,10.19)	0.149
Q2 (11.64–15.10)	2.27 (0.64,8.11)		3.59 (1.34,10.00)	
Q3 (15.10–19.90)	5.33 (1.54,19.42)		4.15 (1.55,11.56)	
Q4 (≥19.90)	6.22 (1.89,21.28)		2.73 (1.02,7.57)	
**Model 2**[c]				
**Dietary Iron (mg/day)**				
Q1 (<11.64)	Reference	0.031	3.64 (1.32,10.44)	0.557
Q2 (11.64–15.10)	2.22 (0.61,8.21)		3.66 (1.33,10.52)	
Q3 (15.10–19.90)	5.60 (1.58,20.87)		4.13 (1.49,11.92)	
Q4 (≥19.90)	7.20 (2.04,26.40)		3.00 (1.05,8.87)	

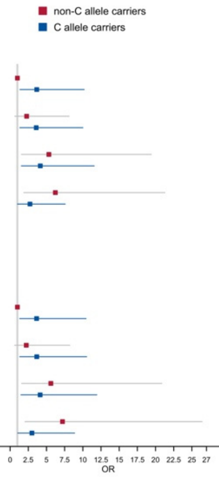

Elevated triglycerides	non-C allele carriers		C allele carriers	
	OR (95%CI)	p trend	OR (95%CI)	p trend
**Model 1**				
**Dietary Iron (mg/day)**				
Q1 (<11.64)	Reference	0.192	10.96 (2.24,198.13)	0.506
Q2 (11.64–15.10)	12.55 (2.09,242.03)		10.78 (2.20,195.00)	
Q3 (15.10–19.90)	10.73 (1.79,206.61)		10.41 (2.12,188.30)	
Q4 (≥19.90)	11.48 (1.96,219.37)		10.30 (2.10,186.29)	
**Model 2**				
**Dietary Iron (mg/day)**				
Q1 (<11.64)	Reference	0.561	11.39 (2.32,206.13)	0.747
Q2 (11.64–15.10)	12.54 (2.07,242.48)		11.03 (2.24,199.75)	
Q3 (15.10–19.90)	11.26 (1.86,217.61)		10.76 (2.17,195.33)	
Q4 (≥19.90)	13.17 (2.18,254.83)		11.08 (2.18,202.57)	

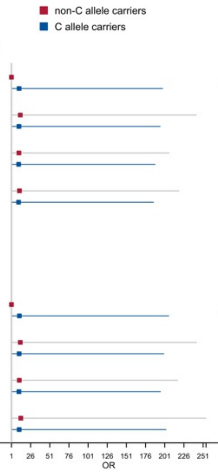

Elevated waist circumference	non-C allele carriers		C allele carriers	
	OR (95%CI)	*p* trend	OR (95%CI)	*p* trend
**Model 1**				
**Dietary Iron (mg/day)**				
Q1 (<11.64)	Reference	0.115	2.39 (1.01,6.18)	0.958
Q2 (11.64–15.10)	3.19 (1.06,10.12)		2.09 (0.88,5.40)	
Q3 (15.10–19.90)	1.28 (0.41,4.08)		2.32 (0.97,5.98)	
Q4 (≥19.90)	3.50 (1.21,10.67)		2.19 (0.92,5.65)	
**Model 2**				
**Dietary Iron (mg/day)**				
Q1 (<11.64)	Reference	0.069	2.38 (1.00,6.15)	0.292
Q2 (11.64–15.10)	3.29 (1.09,10.46)		2.09 (0.88,5.42)	
Q3 (15.10–19.90)	1.39 (0.44,4.44)		2.45 (1.02,6.36)	
Q4 (≥19.90)	4.32 (1.44,13.73)		2.68 (1.09,7.17)	

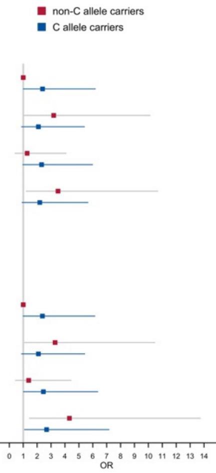

Elevated fasting glucose	non-C allele carriers		C allele carriers	
	OR (95%CI)	*p* trend	OR (95%CI)	*p* trend
**Model 1**				
**Dietary Iron (mg/day)**				
Q1 (<11.64)	Reference	0.612	1.75 (0.63,6.27)	0.395
Q2 (11.64–15.10)	1.05 (0.22,5.02)		1.76 (0.63,6.32)	
Q3 (15.10–19.90)	1.71 (0.44,7.41)		2.64 (0.95,9.42)	
Q4 (≥19.90)	1.42 (0.35,6.22)		2.05 (0.73,7.34)	
**Model 2**				
**Dietary Iron (mg/day)**				
Q1 (<11.64)	Reference	0.984	1.64 (0.58,5.93)	0.310
Q2 (11.64–15.10)	0.96 (0.20,4.65)		1.80 (0.63,6.54)	
Q3 (15.10–19.90)	1.85 (0.47,8.14)		2.65 (0.93,9.59)	
Q4 (≥19.90)	1.61 (0.38,7.40)		2.19 (0.74,8.18)	

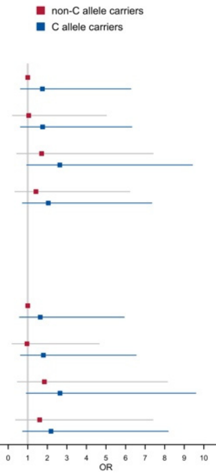

Elevated HDL-C	non-C allele carriers		C allele carriers	
	OR (95%CI)	*p* trend	OR (95%CI)	*p* trend
**Model 1**				
**Dietary Iron (mg/day)**				
Q1 (<11.64)	Reference	0.107	2.82 (0.96,12.06)	0.191
Q2 (11.64–15.10)	1.13 (0.23,6.17)		3.62 (1.23,15.49)	
Q3 (15.10–19.90)	2.66 (0.68,13.23)		2.85 (0.96,12.18)	
Q4 (≥19.90)	2.45 (0.65,11.92)		2.72 (0.92,11.67)	
**Model 2**				
**Dietary Iron (mg/day)**				
Q1 (<11.64)	Reference	0.101	2.63 (0.88,11.30)	0.439
Q2 (11.64–15.10)	1.07 (0.21,5.92)		3.73 (1.26,16.02)	
Q3 (15.10–19.90)	2.93 (0.74,14.70)		2.92 (0.97,12.60)	
Q4 (≥19.90)	2.80 (0.71,14.09)		3.10 (1.00,13.63)	

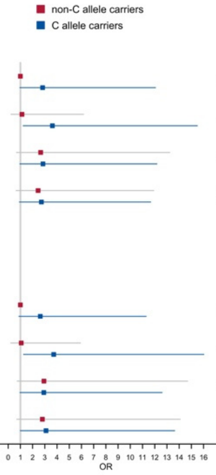

Figures S1–S5: The association between dietary iron and risk of MetS component stratified by C allele presence of the SNP rs2794720 among the female participants.

NOTE: [a] The *p* value for the trend was examined using the medians in each quartile of dietary iron.

[b] Model 1 was adjusted for age, sex, income, education, intentional physical exercise, smoking status, alcohol use, and total dietary energy.

[c] Model 2, in addition to adjusting for the aforementioned covariates, also includes the interaction term between SNP and dietary iron.

	Major allele carriers			Minor allele homozygotes		
	n	OR (95%CI)	p for trend[a]	n	OR (95%CI)	p for trend
**Model 1[b]**						
**Non-heme Iron (mg/day)**						
Q1 (<10.55)	28	Reference	0.115	319	4.53 (1.47,19.92)	0.986
Q2 (10.55–13.81)	34	3.33 (0.74,18.01)		312	3.97 (1.28,17.48)	
Q3 (13.81–18.21)	33	3.24 (0.80,16.65)		313	6.26 (2.03,27.50)	
Q4 (≥18.21)	40	4.67 (1.21,23.33)		206	4.14 (1.34,18.25)	
**Model 2[c]**						
**Non-heme Iron (mg/day)**						
Q1 (<10.55)	28	Reference	0.049	319	4.45 (1.43,19.65)	0.518
Q2 (10.55–13.81)	34	3.18 (0.70,17.28)		312	3.89 (1.25,17.23)	
Q3 (13.81–18.21)	33	3.47 (0.84,17.92)		313	6.19 (1.99,27.44)	
Q4 (≥18.21)	40	5.15 (1.28,26.52)		206	4.60 (1.43,20.76)	

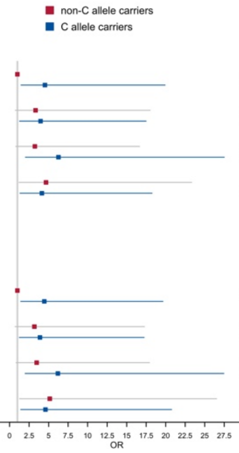

Figure S6: The association between non-heme iron and risk of MetS stratified by C allele presence of the SNP rs2794720 among the female participants.

NOTE: [a] The *p* value for the trend was examined using the medians in each quartile of dietary iron.

[b] Model 1 was adjusted for age, sex, income, education, intentional physical exercise, smoking status, alcohol use, and total dietary energy.

[c] Model 2, in addition to adjusting for the aforementioned covariates, also includes the interaction term between SNP and dietary iron.
